# Induction of CD44 Variant 9-Expressing Cancer Stem Cells Might Attenuate the Efficacy of Chemoradioselection and Worsens the Prognosis of Patients with Advanced Head and Neck Cancer

**DOI:** 10.1371/journal.pone.0116596

**Published:** 2015-03-09

**Authors:** Takeichiro Aso, Mioko Matsuo, Hideyuki Kiyohara, Kenichi Taguchi, Fumihide Rikimaru, Mototsugu Shimokawa, Yuichi Segawa, Yuichiro Higaki, Hirohito Umeno, Tadashi Nakashima, Muneyuki Masuda

**Affiliations:** 1 Department of Head and Neck Surgery, National Kyushu Cancer Center, 3-1-1, Notame, Minamiku, Fukuoka 811-1395, Japan; 2 Department of Pathology, National Kyushu Cancer Center, 3-1-1, Notame, Minamiku, Fukuoka 811-1395, Japan; 3 Department of Cancer Information Research, National Kyushu Cancer Center, 3-1-1, Notame, Minamiku, Fukuoka 811-1395, Japan; 4 Department of Otorhinolaryngology and Head and Neck Surgery, Graduate School of Medical Sciences, Kyushu University, 3-1-1 Maidashi, Higashiku, Fukuoka 812-8582, Japan; 5 Department of Otorhinolaryngology and Head and Neck Surgery, School of Medicine, Kurume University, 67, Asahimachi, Fukuoka, 830-0011, Japan; Karolinska Institutet, SWEDEN

## Abstract

**Background:**

At our institute, a chemoradioselection strategy has been used to select patients for organ preservation on the basis of response to an initial 30–40 Gy concurrent chemoradiotherapy (CCRT). Patients with a favorable response (i.e., chemoradioselected; CRS) have demonstrated better outcomes than those with an unfavorable response (i.e., nonchemoradioselected; N-CRS). Successful targeting of molecules that attenuate the efficacy of chmoradioselection may improve results. Thus, the aim of this study was to evaluate the association of a novel cancer stem cell (CSC) marker, CD44 variant 9 (CD44v9), with cellular refractoriness to chemoradioselection in advanced head and neck squamous cell carcinoma (HNSCC).

**Materials and Methods:**

Through a medical chart search, 102 patients with advanced HNSCC treated with chemoradioselection from 1997 to 2008 were enrolled. According to our algorithm, 30 patients were CRC following induction CCRT and 72 patients were N-CRS. Using the conventional immunohistochemical technique, biopsy specimens and surgically removed tumor specimens were immunostained with the anti-CD44v9 specific antibodies.

**Results:**

The intrinsic expression levels of CD44v9 in the biopsy specimens did not correlate with the chemoradioselection and patient survival. However, in N-CRS patients, the CD44v9-positive group demonstrated significantly (*P* = 0.008) worse prognosis, than the CD44v9-negative group. Multivariate analyses demonstrated that among four candidate factors (T, N, response to CCRT, and CD44v9), CD44v9 positivity (HR: 3.145, 95% CI: 1.235–8.008, *P* = 0.0163) was significantly correlated with the poor prognosis, along with advanced N stage (HR: 3.525, 95% CI: 1.054–9.060, *P* = 0.0228). Furthermore, the survival rate of the CD44v9-induced group was significantly (*P* = 0.04) worse than the CD44v9-non-induced group.

**Conclusions:**

CCRT-induced CD44v9-expressing CSCs appear to be a major hurdle to chemoradioselection. CD44v9-targeting seems to be a promising strategy to enhance the efficacy of chemoradioselection and consequent organ preservation and survival.

## Introduction

Despite recent advances in multidisciplinary treatments, the overall survival and quality of life of patients with advanced head and neck squamous cell carcinoma (HNSCC) have not improved considerably over the past decade [1, 2]. Thus, establishment of clinically effective therapies based on HNSCC biology is imperative. In the Department of Otolaryngology and Head and Neck Surgery at Kyushu University and its affiliated institutes, a strategy called chemoradioselection has been used as a tool to measure the biological aggressiveness of an individual tumor since 1972 [3–5]. In brief, responses of tumors are evaluated following 30–40 Gy of concurrent chemoradiothepapy (CCRT). Then, patients who demonstrate favorable responses (i.e., chemoradioselected; CRS), proceed to further CCRT up to 60–70Gy, whereas those with unfavorable responses (i.e., non-chemoradioselected; N-CRS), undergo radical surgery, which often results in the loss of vital organs (e.g., the larynx). Intriguingly, CRS patients demonstrate significantly better survival and organ preservation irrespective of their clinical stages, suggesting the accuracy of chemoradioselection [3, 5]. Recently, a similar concept of chemoselection was postulated by a group at the University of Michigan, facilitating improved organ preservation and survival [6–8]. Thus, if the efficacy of chemo-/radioselection is enhanced, more improved survival and organ preservation in patients, particularly, those with advanced HNSCC might be feasible [1]. Based on this speculation the aim of this study is to elucidate mechanisms, which attenuate the effects of chemoradioselection to develop clinical effective targeted therapies.

During the last decade, it has become apparent that cancer stem cells (CSCs), which are characterized by strong potential for self-renewal and propagation of heterogeneous tumor, may be the main cause of tumor refractoriness to conventional chemo-/radio therapies [9]. Survival of a single CSC can cause tumor re-growth and more importantly CSCs have been proposed to be a source of distant metastases [9]. In HNSCC, the standard form of CD44 (CD44s) was first identified as a surface marker of CSCs by Prince et al., and it is expressed in < 10% of HNSCC cells [10]. However, the results of an immunohistochemical study, which demonstrated that 60%-95% of cells in the normal epithelium of head and neck and 60%-100% of HNSCC cells expressed CD44s, have cast a doubt on the credibility of this marker [11]. In addition, inoculation of a small number of CD44s-negative HNSCC cells caused development of a bulk tumor in immune-compromised mouse [12, 13]. In contrast, through a series of *in vitro* and *in vivo* assays and experiments with clinical samples, mainly performed in the laboratory of Prof. Saya at Keio University, CD44 variant 9 (CD44v9), a splicing variant of CD44, has emerged as a novel marker of cancer stemness in a variety of solid tumors including HNSCC [14–18]. Functionally, CD44v9 increases the intra-cellular levels of reduced glutathione (GSH) when coupled with xCT, thereby protecting cells from ROS and oxidative stress, which is one of the distinct properties of CSCs [14]. This scenario well explains the mechanism by which CSCs can survive chemo-/radio therapies, because these agents have been reported to exert cytotoxic effects mainly through ROS production of [14, 19]. Indeed, in HNSCC tumor samples, double immunostainings with involucrin, a differentiation marker, and CD44v9 clearly demonstrated a mutually exclusive staining pattern and induction chemotherapy preferentially killed involucrin-positive cancer cells, resulting in the marked induction of CD44v9-positive cells. The expression levels of CD44v9 in HNSCC cell lines were associated with the increased levels of intracellular GHS and resistance to cisplatin. Thus, treatments of CD44v9-expressing HNSCC cell lines with an inhibitor of xCT, sulfasalazine, significantly inhibited cellular viability and tumor growth in nude mice and enhanced sensitivity to cisplatin [16].

In view of these findings, we immunohistochemically examined the expression levels of CD44v9 protein in clinical samples obtained from patients with advanced HNSCC treated according to the platinum-based chemoradioselection strategy to determine if CD44v9-expressing HNSCC cells possess stemness and cause cellular refractoriness to chemoradioselection.

## Materials and Methods

### Patient characteristics, sub-grouping and tissue samples

Through a medical chart search for patients who were treated at our institute from 1997 to 2008, we selected 102 patients to this study who met the following criteria: (1) those with previously untreated hypopharyngeal, laryngeal or oral cavity cancer patients with stage III or IV tumor according to the UICC TNM classification (2002); (2) those treated with the chemoradioselection strategy; (3) those with no distant metastasis; and (4) those with biopsy and/or surgically removed specimens that apparently contained invasive fronts of tumor that were adjacent or surrounded by tumor-associated stroma in our formalin-fixed paraffin-embedded tissue archive; this last criteria was included because scoring of immunostaining was performed in these tumor fronts as described below. The virus-related HNSCCs (i.e., nasopharynx and oropharynx) were excluded from the analyses to focus on the biological role of CD44v9. This study was approved by the Institutional Review Board of the National Kyushu Cancer Center (No 2013-107). Written informed consent was given by participants for their clinical records to be used in this study.

The characteristics of the patients are shown in Table 1. All patients were followed-up for >60 months; the average follow-up period was 51.7 months (range: 2 to 151). Their average age was 60.8 years. There were 27 patients with hypopharyngeal carcinoma, 40 patients with oral carcinoma, and 35 patients with laryngeal carcinoma.

**Table 1:** Patient characteristics (*N* = 102).

	Characteristic	No of patients (%)
Sex	Male	88 (86.3%)
	Female	14 (13.7%)
Subsite	Hypopharynx	27 (36.5%)
	Oral	40 (39.2%)
	Larynx	35 (34.3%)
T stage	T1	11(10.8%)
	T2	29 (28.4%)
	T3	34 (33.3%)
	T4	28 (27.5%)
N stage	N0	16 (15.7%)
	N1	17 (16.7%)
	N2	66 (64.7%)
	N3	3 (2.9%)
Clinical stage	Ⅲ	28 (27.5%)
	Ⅳ	74 (72.5%)

**Fig 1:**
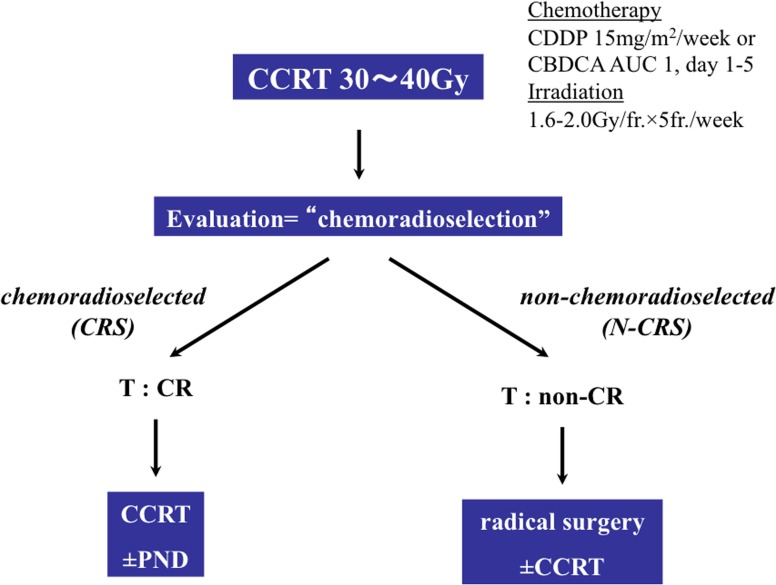
Algorithm-based chemoradioselection treatment protocol. CCRT, concurrent chemoradiotherapy; CDDP, cisplatin; CBDCA, paraplatin; AUC, area under the curve; and PND, planned neck dissection.

The treatment courses of the patients are shown in Fig. 1. Following 30–40 Gy of CCRT comprising cisplatin (CDDP; 15mg/m^2^/day) or parapalatin (CBDCA; AUC = 1/day) from days 1 to 5 and external beam irradiation (2.0 Gy/day) 5days a week, 30 patients were classified into the CRS group (clinical complete response at the primary site) and the remaining 72 patients were classified into the N-CRS group.

After careful examination of the tissue archive, 30 biopsy specimens from N-CRS patients and 30 paired biopsy and surgically removed specimens from the same N-CRS patients were selected. However, the remaining 42 patients in the N-CRS arm did not have proper biopsy specimens that met the criteria mentioned above; therefore only surgically removed tissues were collected from this population. Consequently, a total of 132 (60 biopsy and 72 surgically removed) tissue samples were processed in this study.

### Immunohistochemistry and scoring

**Fig 2:**
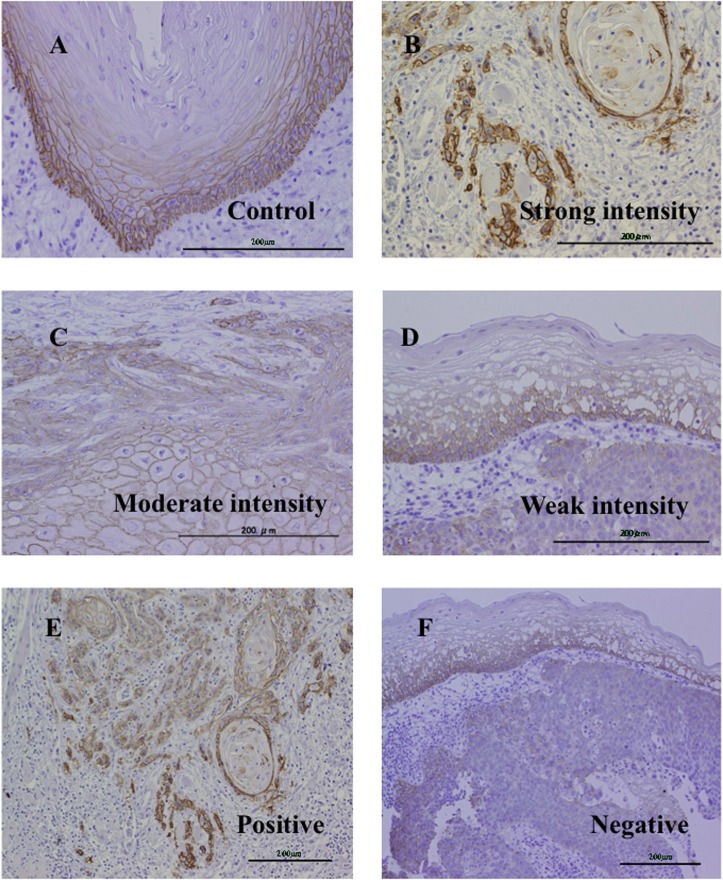
Representative pictures of anti-CD44v9-antibody immunostaining. The staining intensity obtained in the basal cells of normal epithelium was used as a control (A). Tumor samples demonstrated strong (B), moderate (C), and weak (D) intensities relative to the control (A). Respective positive (E) and negative (F) stainings. Bar indicates 200 um.

Anti-human CD44v9 rat IgG monoclonal antibody (RV3), which specifically recognizes human CD44v9, was generated and kindly provided by Prof. Saya, Keio University. This antibody has been used in previous studies [15, 16, 18]. Immunostaining for CD44v9 was performed as described previously [15]. In brief, a VECTASTAIN Elite ABC Standard Kit (Vector Laboratories, Burlingame, CA, USA) with a heated-induced, antigen-retrieval step was used to perform immunohistochemical staining for CD44v9. Xylene was used to deparaffinize the sections, which were rehydrated in a series of ethanols. Heat-induced epitope retrieval was performed in Target Retrieval Solution (S-1699, DAKO, Tokyo, Japan) in an autoclave at 121°C for 15 min. After cooling at room temperature for 20 min, the slides were thoroughly washed in Tris-buffered saline (TBS), pH 7.6. Endogenous peroxidase activity was blocked at room temperature by treatment with 0.3% hydrogen peroxide in methanol for 30 min. The sections were washed in TBS and then transferred to a Shandon Sequenza staining system in a humidified chamber (Thermo Fisher Scientific K.K., Yokohama, Japan). Non-specific antibody binding was inhibited by incubating the sections in 10% normal rabbit serum. The slides were incubated with mouse monoclonal antibody against CD44v9 (RV3) (diluted 1:12500; 0.2μg/ml) at 4°C overnight. These sections were washed thrice with TBS and incubated for 30 minutes in biotinylated rabbit anti-rat IgG (Dako, Tokyo, Japan) diluted 1:200 in Antibody Diluent. The Metal Enhanced DAB Substrate Kit (Thermo Scientific) was used to visualize CD44v9 expression. The slides were counterstained with hematoxylin. Appropriate negative and positive controls were used in each staining run. There were 2 types of negative controls: 1) non-immune rat IgG2a-Negative Isotype control (Millipore, Billerica, MA, USA) with the same concentration as the primary antibody and 2) dilution buffer without the primary antibody. Breast cancer tissue was used as the positive control,

Considering that the basal cells in the normal epithelium of the upper aerodigestive tract show positive staining for CD44v9 (Fig. 2A), counting of CD44v9-positive cells was performed at the invasive fronts of tumors that were adjacent or surrounded by tumor-associated stroma to exclusively count cancer cells. This approach was also based on the speculation that CSCs, including those of HNSCC, frequently reside in the niche located in the tumor-associated stroma [9, 20]. Microscopic analysis was performed by 2 independent observers, including a specialized histopathologist (K.T.) and the average value was adopted for scoring.

The CD44v9 staining score was determined by the sum of the quantity score (i.e., the percentage of positive cancer cells) and the quality score (i.e. the intensity of the staining compared to the staining of normal basal cells) using a method originally proposed by Bankfalvi et al [21]. The quantity scores were defined as follows: 0%, no positive cell; 1, 1%∼25%; 2, 26%∼75%; and 3, 76%∼100%. The quality scores were defined as follows: -1, homogeneously weak staining; 0, heterogeneously similar or strong staining; and 1, homogeneously similar or strong staining. Based on this scoring system, samples with scores from −1∼1 were categorized as CD44v9-negative and samples with scores from 2∼5 were categorized as CD44v9-positive.

### Grading of tumor responses to CCRT

The therapeutic effects of CCRT on the surgical specimens were evaluated according to the criteria defined in the General Rules for Clinical Studies on Head and Neck Cancer (5^th^ Edition) edited by the Japan Society for Head and Neck Cancer. In brief, the effects are classified into 4 grades: Grade 0, no effect; Grade 1, slight effect with ≤1/3 cancer cells still viable; Grade 2, strong effect with 1/3 > cancer cells viable; and Grade 3, complete response with no viable cells.

### Statistical analyses

A Wilcoxon rank sum test was used to analyze the relevance of CD44v9 expression in biopsy specimens to chemoradioselection (i.e., CRS versus N-CRS). We analyzed the correlations between the expression levels of CD44v9 in biopsy specimens and the CD44v9 induction and treatments effects in surgically removed specimens employing a Fisher’s exact test. To compare the disease-specific survival (DSS) rates between the specific groups, Kaplan-Meier curves were generated, and a Wilcoxon test was used to analyze the statistical differences. Univariate and multivariate Cox proportional hazard model were used to calculate the effects of clinicopathlogical factors on DSS rates. Values of *P* < 0.05 were considered statistically significant. All analyses were confirmed by a specialized statistician (M.S).

## Results

### Immunostainings

The representative photos of CD44v9 staining are shown in Fig. 2. The distribution of scores for the 60 biopsy specimens was as follows: score −1, 0; score 0, 13; score 1,15: score 2, 24; score 3, 4; score 4, 4; and score 5, 0. On the other hand, the 72 surgical specimens showed the following distribution: score −1,0; score 0, 11; score, 1,30: score 2, 18; score 3, 13; score 4, 0; and score 5, 0. Consequently, the CD44v9-positive rate was 53% (32/60) for the biopsy specimens, and 43% (31/72) for the surgical specimens. Three primary sites, oral cavity, hypopharynx and larynx, demonstrated similar expression levels of CD44v9 in the biopsy (*N* = 60, *P* = 0.39) and surgically removed (*N* = 72, *P* = 0.092) specimens. When the staining scores were compared between the paired biopsy and surgical specimens obtained from the identical 30 patients, 12 patients demonstrated an increase in CD44v9 expression in the surgical specimens (designated as the CD44v9-induced group), whereas in the remaining 18 patients (designated as the CD44v9-non-induced group), the scores were decreased or unchanged. The paired biopsy specimens were composed of tumors from hypopharynx (9), oral cavity (13), and larynx (9). Every three sites demonstrated similar rates of CD44v9 induction (38–44%).

### Clinical courses, responses to CCRT and postoperative CCRT

**Fig 3:**
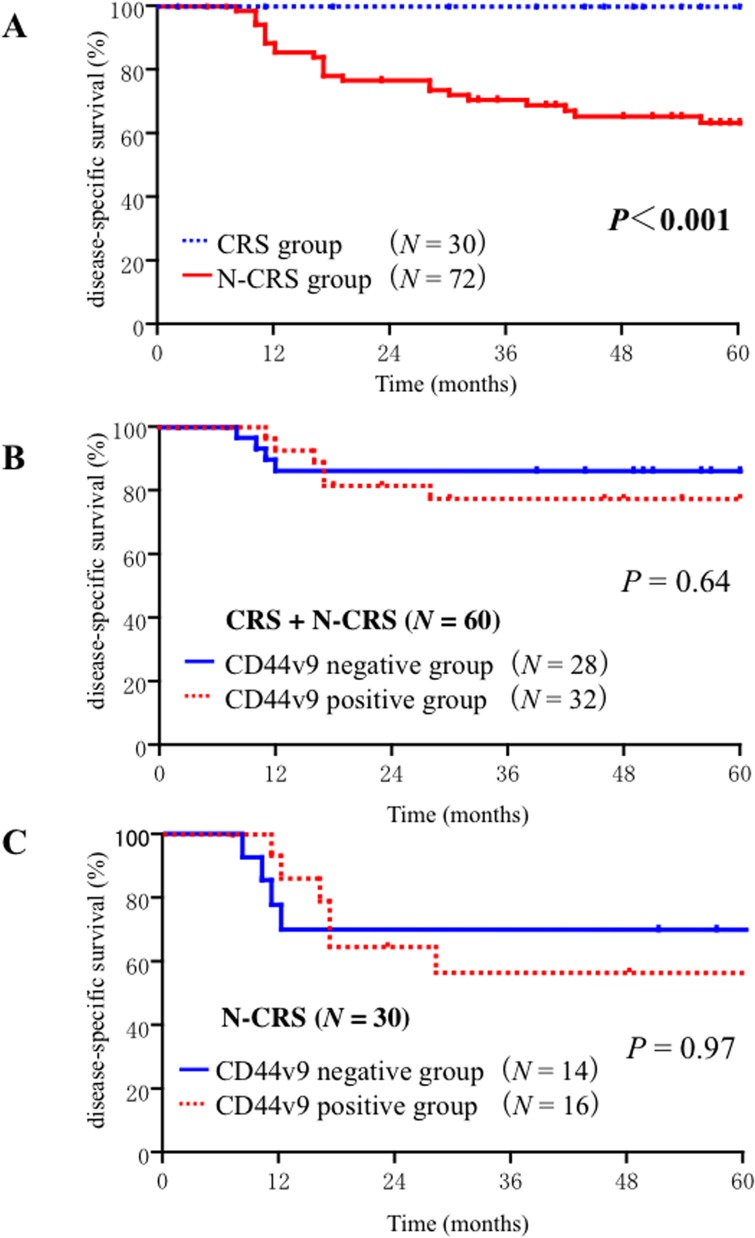
(A) Disease specific survival curves of all patients (n = 102) according to the chemoradioselection. (B) Disease specific survival curves based on the CD44 v9 positivity of biopsy samples (n = 60) obtained from 30 chemoradioselected (CRS) patients and 30 non-chemoradioselected (N-CRS) patients. (C) Disease specific survival curves based on the CD44 v9 positivity of biopsy samples obtained from 30 N-CRS patients.

Kaplan-Meier curves (Fig. 3A) were used to analyze the clinical outcomes of CRS and N-CRS patients (Fig. 1). Consistent with our previous findings, CRS patients demonstrated significantly (*P* < 0.001) better survival [3, 5]. The 72 surgically removed specimens demonstrated G1 (*N* = 58) or G2 (*N* = 14) responses to induction CCRT. Among the 72 N-CRP patients, 54 underwent pot-operative CCRT. No significant difference was observed between the Kaplan-Meier curves of the patients with or without pot-operative CCRT (*P* = 0.507).

### Association of primary tumor site with chemoradioselection and prognosis

We then examined whether the primary tumor site affected the chemoradioselection and patients survival. The cancers of oral cavity demonstrated markedly lower rate of chemoradioselection (1/40, 3%) than oropharynx (14/27, 52%) and larynx (15/35, 42%), consistent with a general consensus that oral cavity cancer is relatively resistant to chemo/radiation among HNSCCs [22]. However, in the Kaplan-Meier analyses of the surgically removed specimen (*N* = 72), the 5-yr DSS rate of oral cavity cancer (50%) was similar (P = 0.80) to those of hypopharynx (65%) and larynx (55%).

### Expression of CD44v9 in the biopsy specimens

To assess the clinical significance of the effect of intrinsic CD44v9 expression on chemoradioselection and patients survival, we compared the expression levels of CD44v9 in the 60 untreated biopsy specimens obtained from CRS (*N* = 30) and N-CRS (*N* = 30) patients. There was no significant difference in CD44v9 expression levels between the CRS and N-CRS samples (*P* = 0.8289). In addition, CD44v9 positivity did not affect Kaplan-Meier DSS curves either in the CRS plus N-CRS cohort (*P* = 0.64; Fig. 3B) or in the N-CRS cohort (*P* = 0.97; Fig. 3C). Similar results were obtained with the univariate Cox proportional hazard model (HR: 1.086, 95% CI: 0.68–1.72; *P* = 0.72). These results suggest that the expression levels of intrinsic CD44v9 in the biopsy specimens are not useful as a predictor of chemoradioselection and the patient survival.

### Expression of CD44v9 in the surgically removed specimens

**Fig 4:**
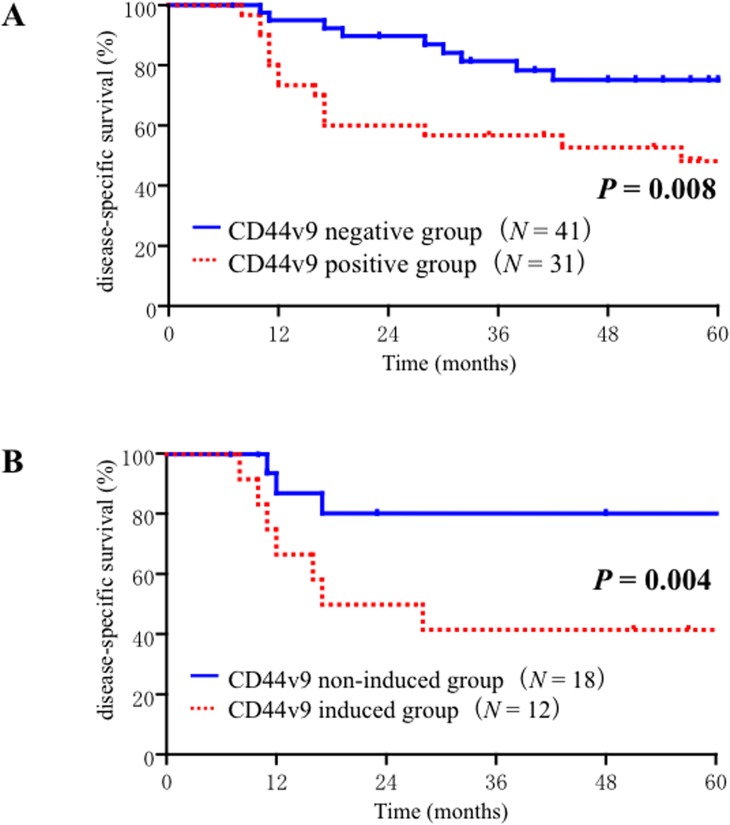
(A) Disease specific survival curves based on the CD44 v9 positivity of surgically removed samples obtained from 72 non-chemoradioselected (N-CRS) patients. (B) Diseasespecific survival curves of 30 N-CRS patients who had paired biopsy and surgically removed samples. The patients were divided into 2 groups according to their levels of CD44v9 expression before and after concurrent chemoradiotherapy.

In view of the above findings, we analyzed whether the expression levels of CCRT-induced CD44v9 were correlated with the unfavorable outcomes in the surgically removed specimens obtained from N-CRS patients. The basis for this analysis was the previous observation that induction chemotherapy apparently enhanced the subset of CD44v9-expressing cells in the HNSCC tumors [16]. In N-CRS patients, the CD44v9-positive group (*N* = 31) demonstrated significantly (*P* = 0.008) worse DSS than the CD44v9-negative group (*N* = 41) (Fig. 4A). Since it was confirmed that the primary tumor site didn’t affect the DSS as mentioned above, we examined the effects of four factors i.e., T, N, tumor responses to CCRT, and CD44v9 positivity on the DSS rate of patients by both univariate and multivariate analyses with a Cox proportional hazard model (Table 2). The univariate analyses demonstrated significantly increased risks of disease-specific death in CD44v9-positive patients (HR: 2.033, 95% CI: 1.071–3.859; *P* = 0.03) and with advanced N (HR: 3.091, 95% CI: 1.045–9.060; *P* = 0.0397). In multivariate analyses, CD44v9 positivity (HR: 3.145, 95% CI: 1.235–8.008, *P* = 0.0163) and advanced N stage (HR: 3.525, 95% CI: 1.054–9.060, *P* = 0.0228) were significantly correlated with poor prognosis (HR: 3.140, 95% CI: 1.230–8.017; *P* = 0.0167), suggesting that among these four factors, CD44v9 expression level is an useful biomarker in the N-CRS population, along with advanced N stage.

**Table 2:** HRs using univariate and multivariate Cox proportional hazard model.

Factor	Class	Univariate analysis			Multivariate analysis		
		HR	95%CI	*P*-value	HR	95%CI	*P*-value
CD44v9	Positive	2.033	1.071–3.859	**0.030**	3.145	1.235–8.008	**0.0163**
	Negative	ref					
T stage	1∼2	ref					
	3∼4	0.827	0.354–1.934	0.6615	1.006	0.426–2.387	0.9887
N stage	0∼1	ref					
	2∼3	3.091	1.054–9.060	**0.0397**	3.525	1.191–10.430	**0.0228**
Treatment effect*	1	1.724	0.514–5.784	0.378	0.837	0.214–3.271	0.7976
	2	ref					

*Criteria defined in the General Rules for Clinical Studies on Head and Neck Cancer (5^th^ Edition) edited by the Japan Society for head and Neck Cancer (for more detail see the Material and Methods).

### Comparison of paired samples

We then analyzed whether the CD44v9-positivity in the biopsy specimen correlated with the induction of CD44v9 in the surgically removed specimens. Intriguingly, the increases of CD44v9 score were observed predominantly (*P* = 0.0236) in patients with CD44v9-negative biopsy specimens (64%, 9/14) than CD44v9-positive patients (19%, 3/16). The expression levels of CD44v9 in the biopsy specimens didn’t correlate with the grading of tumor response to CCRT evaluated in the paired surgically removed specimens (*P* = 0.3992). We further compared DSS curves between the CD44v9-induced group (*N* = 12) and CD44v9-non-induced group (*N* = 18) and found that former had a significantly (p = 0.04) worse DSS rate (Fig. 4B).

Taken together, these results strongly indicated that CCRT-induced CD44v9 expression rather than intrinsic expression is a therapeutic hurdle to chemoradioselection.

## Discussion

During the last decade, the mainstay of treatment for advanced HNSCC has shifted from initial radical surgical resection combined with postoperative radiotherapy to dose-intensified treatment protocols, which are primarily aimed at organ preservation [23, 24]. This trend has been markedly advanced by the recent introduction of CCRT (e.g., clinical trials led by the Radiation Therapy Oncology Group (RTOG)) and sequential therapy comprising induction chemotherapy and CCRT (e.g., Tax 324 protocols), which have resulted in further improvements in organ preservation, locoregional control, and survival [25–28]. Nonetheless, as indicated by recent studies or reviews, these protocols appear to have reached the upper limit of human tolerance of acute and sub-acute toxicities, which have caused frequent laryngoesophageal dysfunction and possible treatment-related deaths [29–31]. Therefore, it appears necessary to decrease the current excessive intensity of treatment for advanced HNSCC by optimizing the therapeutic ratio. On the other hand, we have used a chemoradioselection strategy to avail complete advantages of radical resection and CCRT, while avoiding the severe acute and late toxicities. In our previous studies, CRS patients demonstrated significantly better survival with a functional larynx than N-CRS patients [3, 5], consistent with the findings of the present study. These results suggest that the chemoradioselection strategy may be a promising approach for advanced HNSCC, which can optimize the therapeutic ratio. However, it is obvious that the proportion of CRS patients should be increased to further improve the rates of organ preservation and patient survival. Identifying and targeting molecules that circumvent the effects of chemoradioselection appears to be a highly effective strategy to achieve this goal [1]. As mentioned above, within the current conceptual framework of cancer biology, CSCs are probably the main causes of cellular refractoriness to CCRT; therefore, CSCs are expected to be related to the mechanism that attenuates the efficacy of chemoradioselection [1, 9].

**Fig 5:**
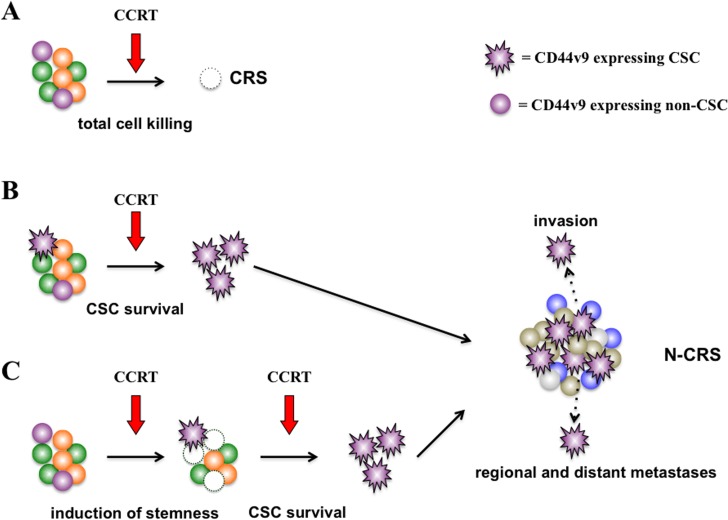
Proposed roles of CD44v9-expressing CSC and non-CSC in the chemoradioselection. (A) CD44v9-expressing non-CSCs are sensitive to CCRT. Intrinsic CD44v9-expressing CSCs (B) or CCRT-induced CD44v9-expressing CSCs (C) can survive CCRT. These CD44v9-expressing CSCs are considered to be highly invasive and metastatic. CSC, cancer stem cell; CCRT, concurrent chemoradiotherapy; CRS, chemoradioselected; and N-CRS, non-chemoradioselected.

In this context, we hypothesized that in advanced HNSCC the expression of a putative CSC marker, CD44v9, may be responsible for the cellular resistance to chemoradioselection. Our data clearly demonstrated that the expression of CD44v9 was correlated with poor outcomes of patients treated with the chemoradioselection strategy, which confirmed our hypothesis. Moreover, we provided the first clinical evidence that CD44v9 may be a useful biomarker and consequently an exploitable molecular target in the treatment of advanced HNSCC. In addition, among other clinicopathological factors that have been used as conventional prognostic markers of HNSCC, the expression of CD44v9 was significantly related to the poor prognosis of patients in multivariate analyses, along with advanced N stage (Table 2). It is of note that CD44v9 demonstrated the lower *P*-value than N stage. Nonetheless, our findings that CCRT-induced CD44v9 expression rather than intrinsic expression had prognostic value should be interpreted carefully. Presumably, CD44v9 expression alone is not sufficient to indicate the property of stemness in cancer cells; CD44v9-expressing cancer cells are likely to be composed of CSCs and non-CSCs. Accordingly, the clinical significance of CD44v9 expression in the chemoradioselection strategy could be explained by at least 3 scenarios, as depicted in Fig. 5. When tumors do not contain CD44v9-expressing CSCs, total cell killing by CCRT is feasible (i.e., CRS; Fig. 5A). On the other hand, when tumors contain CD44v9-expressing CSCs they can survive CCRT (i.e., N-CRS; Fig. 5B). Furthermore, there is a possibility that CCRT, working as a selective pressure, may induce stemness in CD44v9-expressing non-CSCs and lead to cancer cell survival (i.e., N-CRS; Fig. 5C). These selective survivals of CSCs are considered to be sources of local invasion as well as regional and distant metastases, which then worsen the outcomes of N-CRS patients. The previous findings that induction chemotherapy increases the CD44v9-expressing cell population in oral cancer [16], when taken together with our finding that CCRT-induced CD44v9 expression significantly correlates with poor prognosis, support our theory that chemo-/radiotherapy, in a given circumstance, may work as a force of selective sweep or selective pressure that drives HNSCC evolution, leading to the emergence of pluripotent CSCs [1]. These scenarios (Fig. 5) appear to explain the reason why not the intrinsic, but the CCRT-induced CD44v9 expression was useful as a biomarker in our chemoradioselection strategy. In the biopsy specimens, it is not feasible to specifically detect the CD44v9-expressing CSC or CD44v9-expressing non-CSC population that eventually acquire stemness after CCRT: i.e. to distinguish the pattern B and C from A. On the other hand, in the surgically removed samples of the N-CRS patients who underwent CCRT, the CD44v9-expressing cells are supposed to be highly enriched by CSCs, enhancing the value of CD44v9 expression as a biomarker.

Sulfasalazine is a well-characterized specific inhibitor of xCT-mediated cystine transport [17] and is therefore expected to deprive CD44v9-expressing cancer cells from the defense mechanism against ROS. Indeed, administration of sulfasalazine enhanced the intracellular activity of ROS in *in vivo* assays [17] and sensitized HNSCC cell lines to CDDP [16]. Therefore, it is expected that the combination therapy of sulfasalazine and CCRT may significantly enhance the effects of chemoradioselection by sensitizing both intrinsic and CCRT-induced CD44v9-expressing CSCs (Fig. 5A and 5B) to CCRT, and improve the outcomes of patients with advanced HNSCC. Given that sulfasalazine is a commercially available drug that has long been used to treat patients with ulcerative colitis and rheumatoid arthritis, clinical trials of this protocol are now under contemplation.

In conclusion, CD44v9 targeting may provide a new approach to clinically feasible CSC-targeted therapy for HNSCC that can potentiate the efficacy of chemoradioselection and improve organ preservation and survival.

## References

[1] Masuda M, Toh S, Wakasaki T, Suzui M, Joe AK (2013) Somatic evolution of head and neck cancer—biological robustness and latent vulnerability. Mol Oncol 7: 14–28. 10.1016/j.molonc.2012.10.009 23168041 PMC5528403

[2] Leemans CR, Braakhuis BJ and Brakenhoff RH (2010) The molecular biology of head and neck cancer. Nat Rev Cancer 11: 9–22. 10.1038/nrc2982 21160525

[3] Masuda M, Matsuo M, Aso T, Kiyohara H, Rikimaru F, et al. (epub 2014/05/13) Utility of algorithm-based chemoradioselection in the treatment for advanced hypopharyngeal carcinoma. Head Neck. 10.1002/hed.23759 24816950

[4] Masuda M, Kamizono K, Uryu H, Fujimura A, Uchi R (2012) Roles of Therapeutic Selective Neck Dissection in Multidisciplinary Treatment In: Kummoona R., editor editors. Neck dissection—Clinical Application and Recent Advances. In Tech.

[5] Kumamoto Y, Masuda M, Kuratomi Y, Toh S, Shinokuma A, et al (2002) "FAR" chemoradiotherapy improves laryngeal preservation rates in patients with T2N0 glottic carcinoma. Head Neck 24: 637–642. 12112536 10.1002/hed.10114

[6] Worden FP, Kumar B, Lee JS, Wolf GT, Cordell KG, et al (2008) Chemoselection as a strategy for organ preservation in advanced oropharynx cancer: response and survival positively associated with HPV16 copy number. J Clin Oncol 26: 3138–3146. 10.1200/JCO.2007.12.7597 18474879 PMC2742158

[7] Worden FP, Moyer J, Lee JS, Taylor JM, Urba SG, et al (2009) Chemoselection as a strategy for organ preservation in patients with T4 laryngeal squamous cell carcinoma with cartilage invasion. Laryngoscope 119: 1510–1517. 10.1002/lary.20294 19504552 PMC2739984

[8] Urba S, Wolf G, Eisbruch A, Worden F, Lee J, et al (2006) Single-cycle induction chemotherapy selects patients with advanced laryngeal cancer for combined chemoradiation: a new treatment paradigm. J Clin Oncol 24: 593–598. 16380415 10.1200/JCO.2005.01.2047

[9] Visvader JE and Lindeman GJ (2012) Cancer stem cells: current status and evolving complexities. Cell Stem Cell 10: 717–728. 10.1016/j.stem.2012.05.007 22704512

[10] Prince ME, Sivanandan R, Kaczorowski A, Wolf GT, Kaplan MJ, et al (2007) Identification of a subpopulation of cells with cancer stem cell properties in head and neck squamous cell carcinoma. Proc Natl Acad Sci U S A 104: 973–978. 17210912 10.1073/pnas.0610117104 PMC1783424

[11] Mack B, Gires O (2008) CD44s and CD44v6 expression in head and neck epithelia. PLoS One 3: e3360 10.1371/journal.pone.0003360 18852874 PMC2566597

[12] Oh SY, Kang HJ, Kim YS, Kim H, Lim YC (2013) CD44-negative cells in head and neck squamous carcinoma also have stem-cell like traits. Eur J Cancer 49: 272–280. 10.1016/j.ejca.2012.06.004 22770891

[13] Imai T, Tamai K, Oizumi S, Oyama K, Yamaguchi K, et al (2013) CD271 defines a stem cell-like population in hypopharyngeal cancer. PLoS One 8: e62002 10.1371/journal.pone.0062002 23626764 PMC3633921

[14] Nagano O, Okazaki S, Saya H (2013) Redox regulation in stem-like cancer cells by CD44 variant isoforms. Oncogene 32: 5191–5198. 10.1038/onc.2012.638 23334333

[15] Hirata K, Suzuki H, Imaeda H, Matsuzaki J, Tsugawa H, et al (2013) CD44 variant 9 expression in primary early gastric cancer as a predictive marker for recurrence. Br J Cancer 109: 379–386. 10.1038/bjc.2013.314 23778530 PMC3721391

[16] Yoshikawa M, Tsuchihashi K, Ishimoto T, Yae T, Motohara T, et al (2013) xCT inhibition depletes CD44v-expressing tumor cells that are resistant to EGFR-targeted therapy in head and neck squamous cell carcinoma. Cancer Res 73: 1855–1866. 10.1158/0008-5472.CAN-12-3609-T 23319806

[17] Ishimoto T, Nagano O, Yae T, Tamada M, Motohara T, et al (2011) CD44 variant regulates redox status in cancer cells by stabilizing the xCT subunit of system xc(-) and thereby promotes tumor growth. Cancer Cell 19: 387–400. 10.1016/j.ccr.2011.01.038 21397861

[18] Yae T, Tsuchihashi K, Ishimoto T, Motohara T, Yoshikawa M, et al (2012) Alternative splicing of CD44 mRNA by ESRP1 enhances lung colonization of metastatic cancer cell. Nat Commun 3: 883 10.1038/ncomms1892 22673910

[19] Diehn M, Cho RW, Lobo NA, Kalisky T, Dorie MJ, et al (2009) Association of reactive oxygen species levels and radioresistance in cancer stem cells. Nature 458: 780–783. 10.1038/nature07733 19194462 PMC2778612

[20] Krishnamurthy S, Dong Z, Vodopyanov D, Imai A, Helman JI, et al (2010) Endothelial cell-initiated signaling promotes the survival and self-renewal of cancer stem cells. Cancer Res 70: 9969–9978. 10.1158/0008-5472.CAN-10-1712 21098716 PMC3058885

[21] Bankfalvi A, Krassort M, Buchwalow IB, Vegh A, Felszeghy E, et al (2002) Gains and losses of adhesion molecules (CD44, E-cadherin, and beta-catenin) during oral carcinogenesis and tumour progression. J Pathol 198: 343–351. 12375267 10.1002/path.1204

[22] Huang SH, O'Sullivan B (2013) Oral cancer: Current role of radiotherapy and chemotherapy. Med Oral Patol Oral Cir Bucal 18: e233–240. 23385513 10.4317/medoral.18772 PMC3613874

[23] Hanna GJ, Haddad RI, Lorch JH (2013) Induction chemotherapy for locoregionally advanced head and neck cancer: past, present, future? Oncologist 18: 288–293. 10.1634/theoncologist.2012-0286 23442306 PMC3607525

[24] Posner MR (2013) Integrating systemic agents into multimodality treatment of locally advanced head and neck cancer. Ann Oncol 21 Suppl 7: vii246–251. 10.1093/annonc/mdq291 20943623

[25] Posner MR, Hershock DM, Blajman CR, Mickiewicz E, Winquist E, et al (2007) Cisplatin and fluorouracil alone or with docetaxel in head and neck cancer. N Engl J Med 357: 1705–1715. 17960013 10.1056/NEJMoa070956

[26] Vermorken JB, Remenar E, van Herpen C, Gorlia T, Mesia R, et al (2007) Cisplatin, fluorouracil, and docetaxel in unresectable head and neck cancer. N Engl J Med 357: 1695–1704. 17960012 10.1056/NEJMoa071028

[27] Adelstein DJ, Saxton JP, Rybicki LA, Esclamado RM, Wood BG, et al (2006) Multiagent concurrent chemoradiotherapy for locoregionally advanced squamous cell head and neck cancer: mature results from a single institution. J Clin Oncol 24: 1064–1071. 16505425 10.1200/JCO.2005.01.5867

[28] Forastiere AA, Goepfert H, Maor M, Pajak TF, Weber R, et al (2003) Concurrent chemotherapy and radiotherapy for organ preservation in advanced laryngeal cancer. N Engl J Med 349: 2091–2098. 14645636 10.1056/NEJMoa031317

[29] Machtay M, Moughan J, Trotti A, Garden AS, Weber RS, et al (2008) Factors associated with severe late toxicity after concurrent chemoradiation for locally advanced head and neck cancer: an RTOG analysis. J Clin Oncol 26: 3582–3589. 10.1200/JCO.2007.14.8841 18559875 PMC4911537

[30] Argiris A, Brockstein BE, Haraf DJ, Stenson KM, Mittal BB, et al (2004) Competing causes of death and second primary tumors in patients with locoregionally advanced head and neck cancer treated with chemoradiotherapy. Clin Cancer Res 10: 1956–1962. 15041712 10.1158/1078-0432.ccr-03-1077

[31] Corry J, Peters LJ, Rischin D (2010) Optimising the therapeutic ratio in head and neck cancer. Lancet Oncol 11: 287–291. 10.1016/S1470-2045(09)70384-5 20202613

